# Efficacy of Corticosteroids in Patients with SARS, MERS and COVID-19: A Systematic Review and Meta-Analysis

**DOI:** 10.3390/jcm9082392

**Published:** 2020-07-27

**Authors:** Keum Hwa Lee, Sojung Yoon, Gwang Hun Jeong, Jong Yeob Kim, Young Joo Han, Sung Hwi Hong, Seohyun Ryu, Jae Seok Kim, Jun Young Lee, Jae Won Yang, Jinhee Lee, Marco Solmi, Ai Koyanagi, Elena Dragioti, Louis Jacob, Joaquim Radua, Lee Smith, Hans Oh, Kalthoum Tizaoui, Sarah Cargnin, Salvatore Terrazzino, Ramy Abou Ghayda, Andreas Kronbichler, Jae Il Shin

**Affiliations:** 1 Department of Pediatrics, Yonsei University College of Medicine, Seoul 03722, Korea; AZSAGM@yuhs.ac; 2 Yonsei University College of Medicine, Seoul 03722, Korea; sj4068@naver.com (S.Y.); crossing96@yonsei.ac.kr (J.Y.K.); nzsarah61@gmail.com (S.R.); 3 College of Medicine, Gyeongsang National University, Jinju 52727, Korea; gwangh.jeong@gmail.com; 4 Department of Pediatrics, Samsung Changwon Hospital, Sungkyunkwan University School of Medicine, Changwon 51353, Korea; 82agnes@hanmail.net; 5 Department of Global Health and Population, Harvard T.H. Chan School of Public Health, 677 Huntington Avenue, Boston, MA 02115, USA; sunghwihong@gmail.com (S.H.H.); ramy.aboughayda@gmail.com (R.A.G.); 6 Department of Nephrology, Yonsei University Wonju College of Medicine, Wonju 26426, Korea; ripplesong@yonsei.ac.kr (J.S.K.); junyoung07@yonsei.ac.kr (J.Y.L.); kidney74@yonsei.ac.kr (J.W.Y.); 7 Department of Psychiatry, Yonsei University Wonju College of Medicine, Wonju 26426, Korea; jinh.lee95@yonsei.ac.kr; 8 Department of Neuroscience, University of Padova, 35121 Padova, Italy; marco.solmi83@gmail.com; 9 Research and development unit, Parc Sanitari Sant Joan de Déu, CIBERSAM, Dr. Antoni Pujadas, 42, Sant Boi de Llobregat, 08830 Barcelona, Spain; a.koyanagi@pssjd.org (A.K.); louis.jacob.contacts@gmail.com (L.J.); 10 ICREA, Pg. Lluis Companys 23, 08010 Barcelona, Spain; 11 Pain and Rehabilitation Centre, and Department of Health, Medicine and Caring Sciences, Linkoping University, SE-581 85 Linkoping, Sweden; elena.dragioti@liu.se; 12 Faculty of Medicine, University of Versailles Saint-Quentin-en-Yvelines, 78180 Montigny-le-Bretonneux, France; 13 Institut d’Investigacions Biomèdiques August Pi i Sunyer (IDIBAPS), 08036 Barcelona, Spain; radua@clinic.cat; 14 Mental Health Research Networking Center (CIBERSAM), 08036 Barcelona, Spain; 15 Department of Psychosis Studies, Institute of Psychiatry, Psychology and Neuroscience, King’s College London, London SE5 8AF, UK; 16 Centre for Psychiatric Research, Department of Clinical Neuroscience, Karolinska Institutet, 11330 Stockholm, Sweden; 17 The Cambridge Centre for Sport and Exercise Sciences, Anglia Ruskin University, Cambridge CB1 1PT, UK; lee.smith@anglia.ac.uk; 18 School of Social Work, University of Southern California, Los Angeles, CA 90015, USA; hansoh@usc.edu; 19 Department of Basic Sciences, Division of Histology and Immunology, Faculty of Medicine Tunis, Tunis El Manar University, Tunis 1068, Tunisia; kalttizaoui@gmail.com; 20 Department of Pharmaceutical Sciences and Interdepartmental Research Center of Pharmacogenetics and Pharmacogenomics (CRIFF), University of Piemonte Orientale, 28100 Novara, Italy; sarah.cargnin@uniupo.it (S.C.); salvatore.terrazzino@uniupo.it (S.T.); 21 Division of Urology, Brigham and Women’s Hospital and Harvard Medical School, Boston, MA 02115, USA; 22 Department of Internal Medicine IV (Nephrology and Hypertension), Medical University Innsbruck, 6020 Innsbruck, Austria; Andreas.Kronbichler@i-med.ac.at

**Keywords:** corticosteroids, coronavirus, severe acute respiratory syndrome (SARS), Middle East respiratory syndrome (MERS), coronavirus disease 2019 (COVID-19)

## Abstract

(1) Background: The use of corticosteroids in critical coronavirus infections, including severe acute respiratory syndrome (SARS), Middle East Respiratory Syndrome (MERS), or Coronavirus disease 2019 (COVID-19), has been controversial. However, a meta-analysis on the efficacy of steroids in treating these coronavirus infections is lacking. (2) Purpose: We assessed a methodological criticism on the quality of previous published meta-analyses and the risk of misleading conclusions with important therapeutic consequences. We also examined the evidence of the efficacy of corticosteroids in reducing mortality in SARS, MERS and COVID-19. (3) Methods: PubMed, MEDLINE, Embase, and Web of Science were used to identify studies published until 25 April 2020, that reported associations between steroid use and mortality in treating SARS/MERS/COVID-19. Two investigators screened and extracted data independently. Searches were restricted to studies on humans, and articles that did not report the exact number of patients in each group or data on mortality were excluded. We calculated odds ratios (ORs) or hazard ratios (HRs) under the fixed- and random-effect model. (4) Results: Eight articles (4051 patients) were eligible for inclusion. Among these selected studies, 3416 patients were diagnosed with SARS, 360 patients with MERS, and 275 with COVID-19; 60.3% patients were administered steroids. The meta-analyses including all studies showed no differences overall in terms of mortality (OR 1.152, 95% CI 0.631–2.101 in the random effects model, *p* = 0.645). However, this conclusion might be biased, because, in some studies, the patients in the steroid group had more severe symptoms than those in the control group. In contrast, when the meta-analysis was performed restricting only to studies that used appropriate adjustment (e.g., time, disease severity), there was a significant difference between the two groups (HR 0.378, 95% CI 0.221–0.646 in the random effects model, *p* < 0.0001). Although there was no difference in mortality when steroids were used in severe cases, there was a difference among the group with more underlying diseases (OR 3.133, 95% CI 1.670–5.877, *p* < 0.001). (5) Conclusions: To our knowledge, this study is the first comprehensive systematic review and meta-analysis providing the most accurate evidence on the effect of steroids in coronavirus infections. If not contraindicated, and in the absence of side effects, the use of steroids should be considered in coronavirus infection including COVID-19.

## 1. Introduction

Coronavirus disease 2019 (COVID-19), also known as the causative novel virus named severe acute respiratory syndrome coronavirus 2 (SARS-CoV-2), originated in Wuhan, China, at the end of the year 2019 [1,2,3] and subsequently spread across the globe as a pandemic, affecting 213 countries to date [1]. As of 16 May 2020, 4,425,485 confirmed cases have been reported, with a mortality rate of 6.8% (302,059 deaths) [1]. COVID-19 is highly contagious [4,5,6], and there is currently neither a therapeutic agent which showed convincing efficacy, in part due to the inappropriate trial design of most studies [7], nor a vaccine readily available at present time [8,9]. Thus, public health measures have been limited to a focus on curbing the rate of transmission through social distancing.

In the past two decades, the world has already been affected by two severe global human coronavirus (hCoVs) outbreaks: severe acute respiratory syndrome-coronavirus (SARS) [10,11] and Middle East respiratory syndrome-coronavirus (MERS) [12,13], both exhibiting high mortality rates. These two lethal diseases were caused by SARS-CoV in November 2002 [10,14] and MERS-CoV in September 2012 [12,15], respectively. It was only after these outbreaks and studying and treating thousands of confirmed cases that we are now in a position to begin to understand the coronavirus [10,12]. Rapid deterioration or death from the novel coronavirus is in part attributable to the “cytokine storm” caused by the hCoVs [16,17,18], which is the overproduction of immune cells and cytokines (proteins secreted by the immune cells) that surge into the lungs damaging tissue and organs [19,20,21]. Therefore, treatments have involved drugs that regulate this phenomenon, including corticosteroids, for their known properties of immune regulation and their proven efficacy in treating SARS or MERS [16,17].

However, Russell and colleagues worried about the use of corticosteroids in COVID-19 in a recently published paper [22]. The authors stated that steroids may be harmful and therefore should not be used. Furthermore, since there have been no randomized control trials (RCTs), or COVID-19 patient data regarding steroid use until recently, the World Health Organization (WHO) and Center for Disease Control (CDC) do not unreservedly recommend the routine use of corticosteroids [23,24], which obfuscates clinical practices.

Previous systematic reviews in patients with SARS, MERS or COVID-19 have shown conflicting results. Thus, in this systematic review with the meta-analysis of observational studies, we aim to assess the efficacy of corticosteroids for adult patients admitted to hospital with coronavirus disease.

## 2. Methods

### 2.1. Literature Search Strategy and Selection Criteria

For this systematic review and meta-analysis, we followed Preferred Reporting Items for Systematic Reviews and Meta-analyses (PRISMA) guidelines [25] (Table S1). We searched PubMed, MEDLINE, Embase, Web of Science and limited the search to human findings and included reports published in any language. The search terms used were as follows: “MERS”, “middle east respiratory syndrome”, “SARS”, “severe acute respiratory syndrome”, “Coronavirus 19”, “COVID-19”, “SARS-CoV-2”, “2019-nCoV”, “coronavirus”, “steroid”, “corticosteroid”, “glucocorticoid”, “cortisone”, “hydrocortisone”, “prednisone”, “prednisolone”, “dexamethasone”, “triamcinolone” (Table S2). We also extended the references cited in the publications by performing a forward search.

We reviewed papers describing steroids during the extraction process, and, as a result, all-cause mortality was decided as a primary outcome due to data availability. In addition, we also collected data on characteristics of the patients, such as sex or mean age, location and the number of hospitals, type of steroids, duration of steroid use, mean duration between onset of illness and initiation of steroids, and the number of patients with intensive care unit (ICU) care, a mechanical ventilator and acute lung injury (ALI) or acute respiratory distress syndrome (ARDS).

Based on our defined primary outcome of mortality between steroid users and a control group, studies either not reporting the number of patients receiving steroids or mortality rate were excluded even if the use of steroids was described. During the search process, we extended the type of studies not only to RCTs but also prospective cohort, population-based, case-control studies on the association of corticosteroid use and hCoVs. However, case reports, case series and studies without sufficient data were excluded. In this process, we also excluded in vitro or in vivo studies, genetic studies and conference abstracts. The search was restricted to studies in humans.

**Figure 1:**
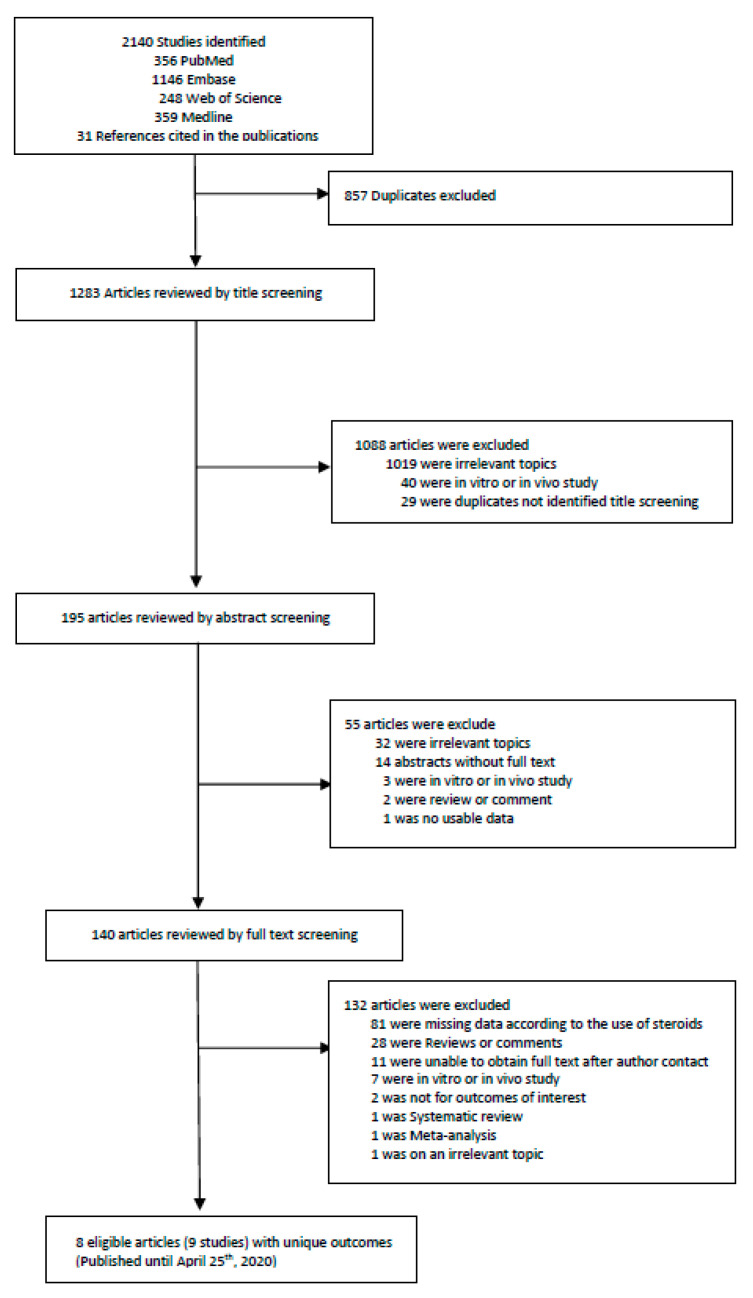
Flow chart of literature search.

Two investigators (KHL and JIS) did the search and manually screened the data. KHL and JIS extracted independently and double-checked to determine whether the eligible articles met the inclusion criteria. The last search was done on 25 April 2020, and we excluded 857 overlapping or duplicated data sets. We first excluded duplicate articles and then labelled all the articles by examining titles, abstracts and full texts in order. From 2140 articles, a total of 8 articles (with 9 studies) were included for the primary outcome. A detailed flow-chart of screening and choosing eligible articles is presented in Figure 1 and Table S3.

### 2.2. Quality Assessment

First of all, we performed the initial quality assessment using A Measurement Tool to Assess Systematic Review 2 (AMSTAR 2) [26] for the systematic review conducted by Russell et al. [22], which is an appraisal tool for systematic reviews (Tables S4 and S5). For this study, we scored one point for each of the fifteen criteria, which the study fully met. If the study did not meet these criteria, or if they did not report the data, we assigned 0 points. Scores of 11–16 were considered high, a moderate score ranged from 6 to 10, and scores between 0–5 were graded low quality.

After the literature search process, we re-assessed the methodological quality of included studies by means of a checklist based on an adapted version of the Newcastle-Ottawa scale [27]. Two reviewers (KHL and JIS) independently performed the quality assessment for included studies. Each study was scored based on an 8-point scale using three criteria: selection of participants (3 points), comparability of studies (1 point), and ascertainment of outcome of interest (4 points). We ranked the studies according to the summed scores: 7 or higher as high quality, moderate quality from 4 to 6 points, and low quality 3 points or less. Two reviewers resolved any discrepancies by re-evaluating the included studies for consensus (Tables S6 and S7).

### 2.3. Analysis of Studies

In this paper, any kind of steroids mentioned in the included studies was accessed. We classified included studies into two categories: (1) intervention and (2) risk factor (Table 1). If the study performed an analysis comparing the steroid group with the non-steroid group as a control, we classified it as “intervention”. These studies were further divided into those that were and were not adjusted for time. Whereas the “intervention” group contained both steroid use alone and steroid as an add-on therapy to ribavirin, other studies included only patients with description of steroid not belonging to the divided group according to steroid use. In this case, it was difficult to match the relationship between corticosteroid itself and mortality because other variables were not corrected. Therefore, it was classified separately into the “risk factor” category, meaning that steroids may act as variables. “ALI/ARDS” was described only in cases where the terms were clearly mentioned according to each definition of the studies. Studies that only described saturation and chest X-ray findings without a diagnosis were not included.

### 2.4. Statistical Analysis

To perform the meta-analysis, MedCalc version 19.2.1 software (MedCalc Software, trial version, Ostend, Belgium) and the statistical software R (version 3.5.1, The R Foundation, Vienna, Austria) were used. Summary effects with 95% confidence interval (CI) and the between-study heterogeneity were estimated. In addition, we performed meta-analyses to calculate the pooled odds ratios (ORs) or hazard ratio (HR) and *p*-value of eligible studies under the fixed and random-effect model to estimate the effect size (ES). *p*-value < 0.05 was considered statistically significant. We also assessed the risk of publication bias with visual inspection of funnel plots and Egger’s asymmetry test [28]. Moreover, *p*-values were defined below thresholds 10^−3^ or 10^−6^ [29,30] and Cochran’s Q test and I^2^ statistic were also calculated for the evaluation of heterogeneity between studies (I^2^ above 50% was considered as serious heterogeneity) [31]. A chi-square test was also performed to analyze the variables which were not sufficient for meta-analysis. In that case, MedCalc version 19.2.1 software was used again and differences were considered significant at *p* < 0.05.

## 3. Results

### 3.1. Included Studies and Baseline Characteristics

We identified eight potentially relevant articles reporting deaths and complications of SARS, MERS and COVID-19 among the steroid group and controls [32,33,34,35,36,37,38,39]. Because one of them [34] was conducted in both Hong Kong and Toronto, the number of total studies actually used in the analysis resulted in nine studies. The characteristics and summary of each of the studies are described in Table 1, Table 2 and Table 3. A detailed description of the included studies are summarized in Tables S8 and S9.

There were seven “intervention” studies: five were not time-adjusted studies (calculated based on ORs without taking into consideration the timing of the event) and two studies were well controlled time-adjusted studies (used Cox regression based on HRs). Steroids were used in more severe cases in three of five non time-adjusted studies. The last two studies were about steroid as a “risk factor”, meaning that steroid users had more severe underlying diseases (Table 1).

The nine studies included five studies investigating outcome in SARS, two in MERS and two in COVID-19. Four of the SARS studies were conducted in China and the other was performed in Canada. In the case of MERS, both studies were conducted in Saudi Arabia. The two COVID-19 studies were both from China. All studies ranged from a single hospital to 14 hospitals combined, and there were also studies that collected patients from all hospitals in the region. The number of patients in each study ranged from 43 to 1743 (Table 1).

Table 2 describes the specific characteristics of the investigated patients. Various kinds and doses of steroids were used, such as methylprednisolone, dexamethasone, hydrocortisone, and prednisolone. Methylprednisolone pulse therapy was used in only two SARS studies within the description permits. The duration of steroid use was noted in one SARS and one MERS study and varied between 3 and 21 days. Overall, there was a delay of about 5–12 days from the onset of symptoms to the start of steroids. Among the included studies, only four studies specifically described ICU treatment. There were three studies that described the number of patients who used ventilators, and one article contained patients with specific diagnoses of ALI or ARDS.

The total number of patients (4051 patients) diagnosed with SARS, MERS, and COVID 19 was 3416, 360, and 275, respectively. Of these, 63.8% of SARS, 43.3% of MERS and 38.9% of COVID-19 patients used steroids. The overall average age of the patients ranged from 40.2 to 58.5 years. In terms of gender distribution, there were more women than men with SARS, while more men were reported to have MERS/COVID-19 (Table 3).

Deaths were mentioned in all included papers as the primary outcome. The total mortality rate of all SARS, MERS and COVID-19 patients was 17.0%, 63.1% and 35.6%, respectively, with or without steroids. In the steroid group, the corresponding figures were: SARS 15.4%, MERS 76.9%, and COVID-19 45.8% (Table 3).

### 3.2. Quality Assessment

A methodological quality assessment of the study conducted by Russell et al. [22] (previously published systematic review on steroid use in hCoVs, influenza and respiratory syncytial viruses (RSV)) was performed according to AMSTAR-2 quality measurement. This study was a simple systematic review without meta-analysis and did not meet the criteria, such as the risk of bias on meta-analysis and publication bias. The final quality score of this article was 2.5 in total showing a low quality compared to our study scored 16 in total (Table S4). We described detailed practical considerations in Table S5.

We re-assessed the quality of included studies based on an adapted version of the Newcastle Ottawa Scale, and found that there were two studies of high quality with a score of above 7 for a total of 8 (Tables S6 and S7). The remaining seven studies were ranked as low quality with a score of 3 points or less. As a result, we performed sensitivity subgroup meta-analyses excluding studies ranked as low quality.

### 3.3. Meta-Analyses

**Figure 2:**
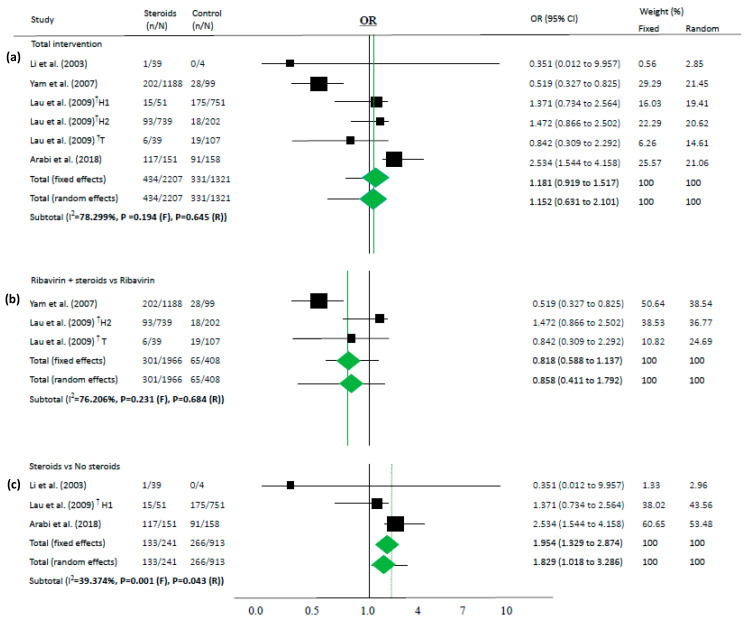
Association between steroids and mortality of studies about “intervention”. Studies are presented as country study (study [year]). The data are presented for total intervention (**a**), steroids as an add-on therapy for ribavirin (**b**), and steroids itself comparing the non-steroid group (**c**). ^†^ These are the same as the paper (Lau (2009) [34]) that has two subgroups: one study conducted in Hong-Kong (H1 and H2) and the other study in Toronto (T).

**Figure 3:**
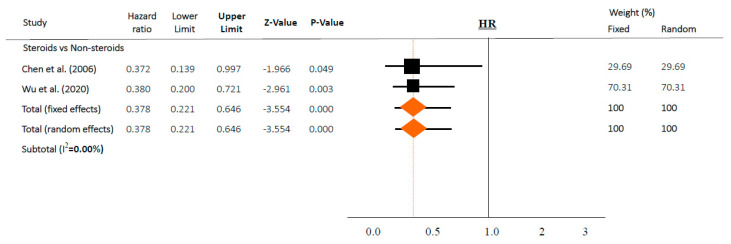
Association between steroids and mortality of studies adjusted for comorbidities or time-dependent conducted studies. Studies are presented as country study (study [year]). Meta-analysis was performed using hazard ratio (HR).

We performed a novel meta-analysis in each subgroup according to the outcome. Pooled ORs for individuals who used steroids (vs. controls) for mortality are presented in Figure 2 as the corresponding forest plots. In two papers in which HR was described [36,37], meta-analysis was performed using HR (Figure 3). The results were described as simple random and fixed-based analysis and 95% CI obtained through meta-analysis. Funnel plots, which are shown in the Supplementary Materials, indicate a risk of publication bias for outcomes (Figures S1–S3).

Because most of the studies showed that the steroid group had more severe cases than the control groups, steroids were not associated with deaths in the overall analysis (OR 1.152, 95% CI 0.631–2.101 in random effects model, I^2^ = 78.3%, Figure 2a). Similarly, in another subgroup analysis, steroids were not associated with mortality among the studies on steroids as an add-on therapy for ribavirin (OR 0.858, 95% CI 0.411–1.792 in random effects model, I^2^ = 76.2%, Figure 2b).

However, in time-adjusted analysis assessing mortality in relation to risk factors such as age or comorbidities, steroids significantly reduced mortality (HR 0.378, 95% CI 0.221–0.646 in random effects model, I^2^ = 0.0%, Figure 3) in two studies [36,37]. In contrast, steroid use and mortality were not significantly associated based on a chi-square test in both studies (*p* = 0.158 and *p* = 0.304, respectively).

**Figure 4:**
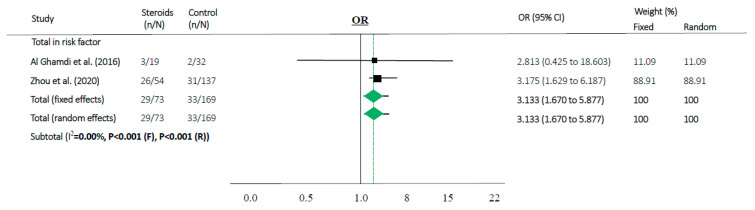
Association between steroids and mortality of studies about “risk factor”. Studies are presented as country study (study [year]). Meta-analysis was performed using an odds ratio (OR).

Steroids were associated with mortality in three studies (OR 1.829, 95% CI 1.018–3.286 in random effects model, I^2^ = 39.37%, Figure 2c). When meta-analyses were performed in studies as risk factors, mortality in MERS and COVID-19 showed high OR (OR 3.133, 95% CI 1.670–5.877 in random effects model, I^2^ = 0.0%, Figure 4).

High heterogeneity (I^2^ > 50%) was observed in the studies of total intervention and steroids as an add-on therapy. Although heterogeneity remained high because of the study characteristics, the *p*-value of heterogeneity was significant for some analyses when subgroup analyses were performed.

### 3.4. Sensitivity Subset Analyses

Sensitivity subset analyses were available for the meta-analyses with high quality (Tables S6 and S7). Interestingly, the scores of included studies with high quality showed a large difference from other studies. In the subset analysis, the result of the outcome was not different from the main meta-analysis (Figure 4). To see how the pathogenicity and risk of mortality induced only by SARS could impact the efficacy of corticosteroids, we performed another subset analysis removing studies about MERS. In this case, subset analyses showed no statistical significance (*p* > 0.05, Figure S4).

## 4. Discussion

To our knowledge, this paper is a comprehensive systematic review and meta-analysis providing the most accurate evidence on steroids as an important treatment of choice in critical coronavirus infections. Until now, there were two other systematic reviews [40,41] dealing with the correlation between corticosteroid and coronavirus; however, one [40] reviewed conducted studies without including time or variable-adjusted statistics and the other [41] only described corticosteroids as an add-on therapy (e.g., combination of ribavirin and corticosteroids). In our study, two reviewers found all the studies from four databases that were missing from the previous study by Russell et al. [22] to avoid selection biases. There had been no statistical analysis on the efficacy of steroids until the current study.

Of note, several included articles in both categories (“intervention” and “risk factors”) should be interpreted with caution. First of all, steroids were used in more severe cases, and the patients chose to enroll in these included studies. Furthermore, studies did not adjust for confounders related to mortality, such as time, age, or comorbidities. As a result, overall, there were no associations between the use of steroids and mortality (OR 1.152, 95% CI 0.631–2.101, Figure 2a). However, after adjusting for time or other variables, steroids significantly reduced the risk of mortality (HR 0.378, 95% CI 0.221–0.646 in random effects model, Figure 3) with similar findings in a fixed-effects model and subset sensitivity analysis. However, when these adjustments were removed after performing a simple chi-square test in both studies, the output was similar to the overall results with no statistically significant differences (*p* = 0.158 and *p* = 0.304, respectively). Taking these results from two well-executed studies [36,37] together, this suggests that well-conducted studies, with time or variable-adjusted statistics, were definitely important in the interpretation of the effect of steroids on lowering mortality. In this regard, prescribing steroids may still be a viable option.

In addition, there were few remaining studies about steroids used in coronavirus after the search process, and most of the studies that ended up being included showed low quality (Tables S6 and S7 and Table 2). Only two enrolled studies [36,37] were ranked as high quality after the search process, and these affected the overall results more than other studies. Excluding low-quality studies changed the final result from no association to better outcome in terms of mortality. We argue that such results from higher quality studies are more appropriate and should be considered relevant before the results of ongoing RCTs become available. As a major point of criticism of the systematic review conducted by Russell et al. [22], the exclusion of high-quality studies can result in the misinterpretation of the currently available evidence.

Moreover, there have been no systematic reviews or meta-analyses on the timing of initiation of steroids in hCoV. Our study is also the first to collect information on the timing of the initiation of steroids. Since several studies did not mention the timing of steroid use in detail, we examined six studies [33,35,36,37,38,39] and found that steroids were more commonly used in patients who had already reached a critically severe status, such as ALI/ARDS or admission to an ICU. In this case, a large amount of steroids was likely required to suppress the “cytokine storm”.

According to our results, the use of steroids in already critically ill patients in the ICU or on mechanical ventilation can make it difficult to prevent disease progression (OR 1.829, 95% CI 1.018–3.286 in random effects model, Figure 2c). Moreover, in our “risk factor” study, people who were older and had more comorbidities used a higher amount of steroids along with progression of the disease and showed high mortality (OR 3.133, 95% CI 1.670–5.877 in random effects model, I^2^ = 0.0%, Figure 4). Therefore, the use of steroids may be important to prevent disease progression because the “cytokine storm” may not be suppressible when the disease is advanced. It can be assumed that if the patient does not have severe symptoms, the use of steroids in low doses may help to treat coronavirus infection without complications.

Discussions on the use of corticosteroids in coronaviruses such as SARS, MERS, and even COVID-19 have been controversial. Recently, Russell et al. performed a systematic review on several studies about the use of steroids in viral infection and cautioned against the use of steroids [22]. However, as a result of examining the quality of studies through AMSTAR2 (Table S4), we found that this paper had potential several critical problems. Among the references cited in this article [22], a selection bias was evident because complications and side effects of steroids were investigated in studies focusing on steroids only without a retrospective control group. For example, studies on complications such as psychosis [42], steroid-induced diabetes mellitus [43], and osteonecrosis [44] only appear in people who used steroids. The inclusion of studies on influenza [45] and RSV [46,47] can also result in selection bias dealing with different types of viruses. As Ioannidis previously suggested [48], if there are selection biases and misinterpretation during the selection of studies, the results can misguide treatments and harm patients.

An important shortcoming is found in Russell et al.’s argument [22] apart from the AMSTAR2 checklist. They did not thoroughly consider the relationship between viral clearance and clinical outcomes. Viral clearance was not significantly associated with clinical outcomes. For SARS, the difference in delayed viral clearance for the treated versus placebo group was two days (17–18 vs. 19–20) [49], and, for MERS, the viral clearance was not significantly associated with 90-day mortality [35]. If the delayed clearance of viral RNA is harmful or not is unclear; however, Russell et al. [22] used it to strengthen their argument.

After Russell et al.’s suggestion, several authors [50,51,52] recommended a short course of corticosteroids at low-to-moderate dose with close monitoring for critically ill patients with COVID-19. Besides, China’s National Health Commission recently developed a modified treatment strategy regarding the use of systematic corticosteroid treatment (methylprednisolone, <1–2 mg per kg body weight, for 3–5 days) for critically ill patients as an adjuvant therapy [53]. However, the WHO [23] and the CDC [24] are yet to change their opinions on the use of steroids based on the study by Russell et al. [22]. For better global guideline about the use of steroids, additional studies are necessary.

There are several potential limitations of our study that should be mentioned. First, all nine included studies were retrospective cohort studies without any RCTs. Because studies depended on the provided records, reporting bias is possible. Moreover, of the 2140 articles on steroids, only two studies [36,37] were adjusted for confounders related to mortality. Until now, there have been no RCTs that have examined the effects of steroids on SARS and MERS based on clinicaltrials.gov, including studies from developing countries. It is surprising that there are no RCTs on this issue for SARS and MERS. As a result, there is a limitation that each result of these included two studies [36,37] have be judged the way it is. Second, there can be potential variations of effects for different combinations of steroids (e.g., patients who received both oral prednisolone and intravenous dexamethasone) or other agents such as antiviral agents or antibiotics. Because the included studies were retrospective studies, meta-analyses could not be performed about this point. Third, confounding factors such as rational variations varied and may bias the data, although we made an effort to minimize this bias. Lastly, we could not perform dose-response meta-analyses between steroids and outcomes because of a lack of data.

Nevertheless, we conducted the systematic review and meta-analyses without missing any of the studies previously published. There has been no meta-analysis and evidence-based statistics related to this topic until now. The results from our study suggest that there is a relationship between steroids and better outcomes, especially in the well-controlled studies even though they were not RCTs. This can also be an important key for treatment of COVID-19. In the United Kingdom, the RECOVERY trial recruited 2104 patients in the steroid arm and has recently concluded that dexamethasone in a dosage of 6 mg daily reduced 28-day mortality among patients receiving invasive mechanical ventilation or oxygen at randomization (RR 0.65, 95% CI 0.51–0.82, *p* < 0.001; RR 0.80, 95% CI 0.70–0.92; respectively) [54]. These preliminary findings confirm a report of efficacy of dexamethasone in the management of ARDS [55]. Other RCTs in COVID-19 have also been registered at clinicaltrials.gov (NCT04244591, NCT04263402, NCT04273321 and NCT04348305) so far and these RCTs are expected to lead to more accurate results on the efficacy of corticosteroids.

## 5. Conclusions

In conclusion, our systematic review and meta-analysis found that steroid use can be beneficial in coronavirus infections. Most studies have initiated steroids late and in more severely affected individuals, clearly indicating a selection bias. The use of such low-quality studies in a systematic review can be misleading and could influence treatment strategies issued by organizations such as the WHO or the CDC and can potentially withhold efficient therapies. Based on the results of high quality and time-dependent studies included in our work, which was adjusted for confounders and comorbidities and showed significant relations between steroid use and better outcome in critical coronavirus infection, we suggest that policymakers communicate the existing state of evidence to practitioners regarding steroid use.

These findings highlight that steroids can potentially be a good therapeutic weapon to overcome coronavirus infection. Considering the results to come from the RECOVERY trial, this methodological analysis is likely the main strength of this paper. Further RCTs will be necessary in the future, but we believe that our meta-analyses can provide very important insights for the present pandemic.

## Figures and Tables

**Table 1:** Main characteristics and findings of the studies included in the meta-analysis.

Authors	Disease	Location/Country	Hospitals, *n*	Sample Size, *n*	Patients Used Steroids, *n*	Controls, *n*	Deaths in Steroid Group, *n* (%)	Deaths in Controls, *n* (%)	Description	Comment
**Intervention (OR, not-adjusted time): steroids were used in more severe cases in 3/5 included studies**										
**Li et al. (2003)** [32]	SARS	Beijing/China	1	43	39	4	1 (2.6)	0 (0.0)	Steroid (*n* = 39 (91%)); Non-steroid (*n* = 4 (9%))	MP was effective for SARS patients.
**Yam et al. (2007)** [33]	SARS	Hong Kong/China	14	1287	1188	99	202 (17.0)	28 (28.3)	Ribavirin + Steroid (*n* = 1188 (92%)); Ribavirin (*n* = 99 (8%))	Ribavirin + Steroid group received more ICU care than control
**Lau et al. (2009)_H ^†^** [34]	SARS	Hong Kong/China	-	1743	51	751	15 (1.9)	175 (18.4)	Steroid (*n* = 51 (3%)); No therapy (*n* = 751 (43%)]	Steroid group was older and had more comorbidities than control
					739	202	93 (11.8)	18 (1.9)	Ribavirin + Steroid (*n* = 739 (42%)); Ribavirin (*n* = 202 (12%))	
**Lau et al. (2009)_T ^†^** [34]	SARS	Toronto/Canada	-	191	42 ^‡^	149 ^‡^	6 (14.3)	19 (12.8)	Ribavirin + Steroid (*n* = 39 (42%)); Ribavirin (*n* = 107 (56%))	Longer delay between symptom onset and admission, hazy chest radiograph were more likely to be treated with combination therapy
Arabi et al. (2018) * [35]	MERS	All/Saudi Arabia	14	309	151	158	117 (77.5)	91 (57.6)	Steroid (*n* = 151 (49%)); Non-steroid (*n* = 158 (51%))	Steroid was not associated 90-day mortality; Steroid group is more severe than control group
**Intervention (HR, adjusted time): adjusted for comorbidities or time-dependent conducted studies**										
**Chen et al. (2006)** [36]	SARS	Guangzhou/China	-	152	121	31	18 (14.9)	7 (22.6)	Steroid (*n* = 121 (80%)); Non-steroid (*n* = 31 (20%)) HR 0.372 (95% CI 0.139–0.998) among critical patients in ICU (*n* = 152)	Steroid significantly reduced the case fatality among critical SARS after death-related variables were adjusted
**Wu et al. (2020)** [37]	COVID-19	Wuhan/China	1	84 ^§^	50 ^§^	34 ^§^	23 (46.0) ^§^	21 (61.8) ^§^	Steroid (*n* = 62 (31%)]: Non-steroid (*n* = 139 (69%)) HR 0.38 (95% CI 0.20–0.72) among patients with ARDS (*n* = 201)	MP decreased the risk of death among patients with ARDS
**Risk factor (not-adjusted time): steroid users had more severe underlying diseases**										
**Al Ghamdi et al. (2016)** [38]	MERS	Jeddah/Saudi Arabia	1	51	5	46	3 (60.0)	16 (34.8)	Survival (*n* = 2/32 (6%)); Death (*n* = 3/19 (16%)): steroid-used patients/each groups (*p* = 0.348)	All deaths received ICU care and all survivors were non ICU patients; non-survivors were more severe
**Zhou et al. (2020)** [39]	COVID-19	Wuhan/China	2	191	57	134	26 (45.6)	28 (20.9)	Survival (*n* = 31/137 (23%)); Death (*n* = 26/54 (48%)): steroid-used patients/each groups (*p* = 0.0005)	Steroid was used in both groups after ARDS; Non-survivors were older and had more comorbidities than survivors

No: Number, SARS: Severe acute respiratory syndrome, MERS: Middle East respiratory syndrome, COVID-19: Coronavirus disease 19, MP: Methylprednisolone, Pulse: High-dose intravenous, methylprednisolone therapy, OR: Odds ratio, HR: Hazard ratio, CI: Confidence interval, ICU: Intensive care unit, ARDS: Acute respiratory distress syndrome, (-): no information. * This paper is also described by Russell (2020) [22] as references. ^†^ These are the same paper (Lau (2009) [34]) that has two subgroups: one study conducted in Hong-Kong (H) and the other study in Toronto (T). ^‡^ These numbers also contain patients with neither treatment or steroids alone. ^§^ All enrolled patients here were diagnosed with ARDS.

**Table 2:** Detailed distribution of patient of the studies included in the meta-analysis.

Authors	Patient Group	Mean Age (SD), Years	Male: Female	Type of Steroids	Duration of Steroids, Day	Mean Duration between Onset of Illness and Steroid Initiation, Day (SD)	ICU Care in Steroids	ICU Care in Controls	Ventilator in Steroids	Ventilator in Controls	ALI/ARDS in Steroids	ALI/ARDS in Controls	Quality Assessment ^‡^
**Intervention (OR, not adjusted time)**													
**Li et al. (2003)** [32]	Non-ICU	-	-	MP, pulse	-	-	0	0	0	0	-	-	Low
**Yam et al. (2007)** [33]	ICU/Non-ICU	-	553:734	HC, MP, PL, pulse	15–21	5 (1)	243	4	161	4	7	49	Low
**Lau et al. (2009)_H ^†^** [34]	ICU/Non-ICU	-	773:970	CS	-	-	-	-	-	-	-	-	Low
**Lau et al. (2009)_T ^†^** [34]	ICU/Non-ICU	-	74:117	CS	-	-	-	-	-	-	-	-	Low
Arabi et al. (2018) * [35]	ICU	-	213:96	HC, DX, MP, PL	3–21	10 (3)	151	158	141	121	-	-	Low
**Intervention (HR, adjusted time)**													
**Chen et al. (2006)** [36]	ICU	40.2(14.6)	68:84	HC, MP, PL	-	4.9 (3.6)	152	0	-	-	-	-	High
**Wu et al. (2020)** [37]	ICU	58.5	60:24	MP	-	-	-	-	-	-	-	-	High
**Risk factor (not adjusted time)**													
**Al Ghamdi et al. (2016)** [38]	ICU/Non-ICU	54	40:11	HC	-	-	3	16	-	-	-	-	Low
**Zhou et al. (2020)** [39]	ICU/Non-ICU	56	119:72	CS	-	12 (4)	-	-	-	-	-	-	Low

SD: Standard deviation, No: Number, ICU: Intensive care unit, CS: Simply stated as corticosteroid in the manuscript, MP: Methylprednisolone, PL: Prednisolone, Pulse: High-dose intravenous methylprednisolone therapy, HC: Hydrocortisone, DX: Dexamethasone, ALI: Acute lung injury, ARDS: Acute respiratory distress syndrome, OR: Odds ratio, HR: Hazard ratio, (-): no information. * This paper is also described by Russell (2020) [22] as references. ^†^ These are the same as the paper (Lau (2009) [34]) that has two subgroups: one study conducted in Hong-Kong (H) and the other study in Toronto (T). ^‡^ The quality of included studies was assessed using an adapted version of the Newcastle Ottawa Scale [27]. See Tables S6 and S7.

**Table 3:** Summary of the characteristics of the included studies.

Variables	SARS *n* (%)	MERS, *n* (%)	COVID-19, *n* (%)
**No. of patients**	3416	360	275
**Steroid used**	2180 (63.8)	156 (43.3)	107 (38.9)
**control**	1236 (36.2)	204 (56.7)	168 (61.1)
Sex *			
**Male**	1468	253	179
**Female**	1905	107	96
**Country**			
**China**	3225 (94.4)	-	275 (100)
**Canada**	191 (5.6)	-	-
**Saudi-Arabia**	-	360 (100)	-
Onset of steroid *	2	1	1
**Early (<7 days from onset of illness)**	2	0	0
**Late (>7 days from onset of illness)**	0	1	1
ICU care *	399	19	-
**Steroid group**	395	3	-
**Controls**	4	16	-
Ventilator *	165	262	-
**Steroid group**	161	141	-
**Controls**	4	121	-
ALI/ARDS *	56	-	-
**Steroid group**	7	-	-
**Controls**	49	-	-
**Deaths**	582	227	98
**Steroid group**	335 (57.6)	120 (52.9)	49 (50.0)
**Controls**	247 (42.4)	107 (47.1)	49 (50.0)
**Deaths/No.**	582/3416 (17.0)	227/360 (63.1)	98/275 (35.6)
**Steroid group**	335/2180 (15.4)	120/156 (76.9)	49/107 (45.8)
**Controls**	247/1236 (20.0)	107/204 (52.5)	49/168 (29.2)

No: Number, SARS: Severe acute respiratory syndrome, MERS: Middle East respiratory syndrome, COVID-19: Coronavirus disease 19, ICU: Intensive care unit, ALI: Acute lung injury, ARDS: Acute respiratory distress syndrome, (-): no information. * The number of studies that report the onset of steroids for SARS and MERS, respectively.

## Supplementary Materials

The following are available online at https://www.mdpi.com/2077-0383/9/8/2392/s1, Figure S1: Funnel plot for meta-analysis of association between steroids and mortality of studies about intervention (in total), Figure S2. Funnel plot for meta-analysis of association and mortality of studies about steroids as an add-on therapy for ribavirin, Figure S3. Funnel plot for meta-analysis of association between steroids and mortality of studies about steroids itself comparing non-steroid group, Figure S4: Association between steroids and mortality of SARS studies about intervention, Table S1: Checklist summarizing compliance with PRISMA guidelines1, Table S2: Search strategy, Table S3: Reason for exclusion during full text screening, Table S4: AMSTAR2 checklist- Quality assessment† for systematic review of Lancet article, Table S5: Summary of practical considerations from Table S4, Table S6: Quality assessment* of the cohort studies included in the meta-analysis (selection part), Table S7: Quality assessment* of the cohort studies included in the meta-analysis (Comparability/Outcome part), Table S8: Detailed description about basal characteristics of included studies, Table S9: Detailed description about steroids of included studies.
