# Supplementary material for: Efficacy of Corticosteroids in Patients with SARS, MERS and COVID-19: A Systematic Review and Meta-Analysis
Source: J Clin Med. 2020 Jul 27;9(8):2392. doi: 10.3390/jcm9082392 (PMC7465945; doi:10.3390/jcm9082392)
Supplement: Supplementary file 1 [file jcm-09-02392-s001.pdf]

Supplementary Appendix
: ‘Efficacy of corticosteroids in SARS, MERS and COVID-19: A systematic review and meta-analysis’

Table of contents

1. PRISMA Checklist 1
2. Search strategy 3
3. Reasons for study exclusion..4
4. Quality Assessment for systematic review..5
5. Quality Assessment of the included Studies..8
6. Detailed distribution of patients on the studies included studies..12
7. Funnel plots 16
8. Subset analyses..18
9. References 19

1. PRISMA Checklist

Table S1. Checklist summarizing compliance with PRISMA guidelines [1].

Section/Topic	#	Checklist Item	Reported on Page #
**TITLE**			
Title	1	Identify the report as a systematic review, meta-analysis, or both.	Title
ABSTRACT			
Structured summary	2	Provide a structured summary including, as applicable: background; objectives; data sources; study eligibility criteria, participants, and interventions; study appraisal and synthesis methods; results; limitations; conclusions and implications of key findings; systematic review registration number.	2
INTRODUCTION			
Rationale	3	Describe the rationale for the review in the context of what is already known.	2-3
Objectives	4	Provide an explicit statement of questions being addressed with reference to participants, interventions, comparisons, outcomes, and study design (PICOS).	2-3
METHODS			
Protocol and registration	5	Indicate if a review protocol exists, if and where it can be accessed (e.g., Web address), and, if available, provide registration information including registration number.	N/A
Eligibility criteria	6	Specify study characteristics (e.g., PICOS, length of follow-up) and report characteristics (e.g., years considered, language, publication status) used as criteria for eligibility, giving rationale.	3-4
Information sources	7	Describe all information sources (e.g., databases with dates of coverage, contact with study authors to identify additional studies) in the search and date last searched.	3-4
Search	8	Present full electronic search strategy for at least one database, including any limits used, such that it could be repeated.	3-4
Study selection	9	State the process for selecting studies (i.e., screening, eligibility, included in systematic review, and, if applicable, included in the meta-analysis).	3-4 (Figure1)
Data collection process	10	Describe method of data extraction from reports (e.g., piloted forms, independently, in duplicate) and any processes for obtaining and confirming data from investigators.	3-4
Data items	11	List and define all variables for which data were sought (e.g., PICOS, funding sources) and any assumptions and simplifications made.	3-4
Risk of bias in individual studies	12	Describe methods used for assessing risk of bias of individual studies (including specification of whether this was done at the study or outcome level), and how this information is to be used in any data synthesis.	3-4, suppl 7-8

Summary measures	13	State the principal summary measures (e.g., risk ratio, difference in means).	3-5
Synthesis of results	14	Describe the methods of handling data and combining results of studies, if done, including measures of consistency (e.g., I^2) for each meta-analysis.	5

Section/Topic	#	Checklist Item	Reported on Page #
Risk of bias across studies	15	Specify any assessment of risk of bias that may affect the cumulative evidence (e.g., publication bias, selective reporting within studies).	9
Additional analyses	16	Describe methods of additional analyses (e.g., sensitivity or subgroup analyses, meta-regression), if done, indicating which were pre-specified.	N/A
RESULTS			
Study selection	17	Give numbers of studies screened, assessed for eligibility, and included in the review, with reasons for exclusions at each stage, ideally with a flow diagram.	5-11
Study characteristics	18	For each study, present characteristics for which data were extracted (e.g., study size, PICOS, follow-up period) and provide the citations.	5-11, Table 3, suppl 9-10
Risk of bias within studies	19	Present data on risk of bias of each study and, if available, any outcome level assessment (see item 12).	9
Results of individual studies	20	For all outcomes considered (benefits or harms), present, for each study: (a) simple summary data for each intervention group (b) effect estimates and confidence intervals, ideally with a forest plot.	5-11, Supplementary tables
Synthesis of results	21	Present results of each meta-analysis done, including confidence intervals and measures of consistency.	5-11
Risk of bias across studies	22	Present results of any assessment of risk of bias across studies (see Item 15).	9
Additional analysis	23	Give results of additional analyses, if done (e.g., sensitivity or subgroup analyses, meta-regression [see Item 16]).	N/A
DISCUSSION			
Summary of evidence	24	Summarize the main findings including the strength of evidence for each main outcome; consider their relevance to key groups (e.g., healthcare providers, users, and policy makers).	11-13
Limitations	25	Discuss limitations at study and outcome level (e.g., risk of bias), and at review-level (e.g., incomplete retrieval of identified research, reporting bias).	11-13
Conclusions	26	Provide a general interpretation of the results in the context of other evidence, and implications for future research.	13
FUNDING			
Funding	27	Describe sources of funding for the systematic review and other support (e.g., supply of data); role of funders for the systematic review.	Title page

2. Search strategy

We searched PubMed, MEDLINE, Embase, Web of Science for reports published in any language until 25 April 2020, that assessed the association between steroids and SARS/MERS. We searched all fields for coronavirus (search terms:”coronavirus”, "SARS”,"severe acute respiratory syndrome,”MERS”,”middle east respiratory syndrome”,“Coronavirus 19”, “COVID-19”, “SARS-CoV-2”, “2019-nCoV”) and terms of various kinds of steroids (search terms:”steroid*”, “corticosteroid*”, "glucocorticoid", "cortisone","hydrocortisone", "prednisone",”prednisolone”, "dexamethasone","triamcinolone").

Full search strategies for each database are given in Table S2.

Table S2. Search strategy.

Database	Number of Studies	Search Terrns
PubMed	356	("MERS” OR “middle east respiratory syndrome” OR "SARS" OR "severe acute respiratory syndrome" OR “Coronavirus 19” OR “COVID-19” OR “SARS-CoV-2” OR “2019-nCoV” OR “coronavirus")<br>AND<br>(“steroid*” OR “corticosteroid*” OR "glucocorticoid" OR "cortisone" OR "hydrocortisone" OR "prednisone" OR “prednisolone” OR "dexamethasone" OR "triamcinolone")
Medline	359	("MERS” OR “middle east respiratory syndrome” OR "SARS" OR "severe acute respiratory syndrome" OR “Coronavirus 19” OR “COVID-19” OR “SARS-CoV-2” OR “2019-nCoV” OR “coronavirus")<br>AND<br>(“steroid*”OR “corticosteroid*” OR "glucocorticoid" OR "cortisone" OR "hydrocortisone" OR "prednisone" OR “prednisolone” OR "dexamethasone" OR "triamcinolone")
Embase	1146	(MERS OR ‘middle east respiratory syndrome’ OR SARS OR ‘severe acute respiratory syndrome’ OR ‘Coronavirus 19’ OR ‘COVID-19’ OR ‘SARS-CoV-2’ OR ‘2019-nCoV’ OR coronavirus)<br>AND<br>(steroid* OR corticosteroid* OR glucocorticoid OR cortisone OR hydrocortisone OR prednisone OR prednisolone OR dexamethasone OR triamcinolone)
Web of Science	248	((TS = MERS) OR (TS = middle east respiratory syndrome) OR (TS = SARS) OR (TS = severe acute respiratory syndrome) OR (TS = Coronavirus 19) OR (TS = COVID-19) OR (TS = SARS-CoV-2) OR (TS = 2019-nCoV) OR (TS = coronavirus))<br>AND<br>((TS = steroid*)OR (TS = corticosteroid*) OR (TS = glucocorticoid) OR (TS = cortisone) OR (TS = hydrocortisone) OR (TS = prednisone) OR (TS = prednisolone) OR (TS = dexamethasone) OR (TS = triamcinolone))

3. Reasons for study exclusion

We manually screened the retrieved articles which were met inclusion criteria. After excluding studies by examining titles and abstracts, full texts of 140 studies were eligible for inclusion. 132 studies were retrieved following reasons**:**

Table S3. Reason for exclusion during full text screening.

Number of Studies	Reason
81	Missing data on death or complication according to the use of steroids
28	Reviews or comments
11	Not accessible full paper even from the homepage of journal or authors
7	In vivo or in vitro studies
2	Not for outcomes of interest
1	Systematic review [2]
1	Meta-analysis [3]
1	Irrelevant topics *

* Not study for coronavirus including SARS, MERS and COVID-19 (SARS: Severe acute respiratory syndrome, MERS: Middle East respiratory syndrome, COVID-19: Coronavirus disease 19).

4. Quality Assessment for systematic review (Table S4–5)

Table S4. AMSTAR2 checklist- Quality assessment† for systematic review of Lancet article [4] *.

Title	Checklist items	Russell et al. (2020) [4] *	Our study
		**Score**	**Score**
1.Question and Inclusion	Did the research questions and inclusion criteria for the review include the components of PICO?‡	0	1
2.Protocol	Did the report of the review contain an explicit statement that the review methods were established prior to the conduct of the review and did the report justify any significant deviations from the protocol?	0	1
3.Study Design	Did the review authors explain their selection of the study designs for inclusion in the review?	0	1
4.Comprehensive Search	Did the review authors use a comprehensive literature search strategy?	0	1
5.Study selection	Did the review authors perform study selection in duplicate?	0	1
6.Data Extraction	Did the review authors perform data extraction in duplicate?	0	1
7.Excluded Studies Justification	Did the review authors provide a list of excluded studies and justify the exclusions?	0	1
8.Included Studies Details	Did the review authors describe the included studies in adequate detail?	0.5	1
9.RoB	Did the review authors use a satisfactory technique for assessing the risk of bias (RoB) in individual studies that were included in the review?	0	1
10.Funding Sources	Did the review authors report on the sources of funding for the studies included in the review?	1	1
11.Statistical Methods	If meta-analysis was performed did the review authors use appropriate methods for statistical combination of results?	0	1
12.RoB on meta – analysis	If meta-analysis was performed, did the review authors assess the potential impact of RoB in individual studies on the results of the meta-analysis or other evidence synthesis?	-	1
13.RoB in individual Studies	Did the review authors account for RoB in individual studies when interpreting/ discussing the results of the review?	0	1

14.Explanation for Heterogeneity	Did the review authors provide a satisfactory explanation for, and discussion of, any heterogeneity observed in the results of the review?	0	1
15.Publication Bias	If they performed quantitative synthesis did the review authors carry out an adequate investigation of publication bias (small study bias) and discuss its likely impact on the results of the review?	-	1
16.Conflict of Interest	Did the review authors report any potential sources of conflict of interest, including any funding they received for conducting the review?	1	1
Total Score		2.5	16
Quality of assessment		Low	High

RoB: risk of bias, COVID-19: coronavirus 2019, WHO: World Health Organization, CDC: Centers for Disease Control and Prevention. *Russell et al. (2020) [4] is a recent systematic review which is against to use corticosteroids in COVID-19. WHO and CDC cited this article and made a current guidance about not-recommending to use steroids in COVID-19. † Quality assessment scoring: 1=study met the criteria (Yes); 0.5= study partially met the criteria (Partial Yes); 0=study did not meet the criteria, or not reported (No); (-) = Not meta-analysis conducted. Scores of 11-16 were considered high, moderate was scored range from 6 to 10 and were scores of 0–5 were graded low quality. ‡ PICO: Population, Intervention, Comparator group, Outcome.

Table S5. Summary of practical considerations from Table S4.

Title of Checklist Items	Considerations	Result
Russell et al. (2020) [4]		
Comprehensive Search	There was no comprehensive literature search strategy in the manuscript	Most of coronavirus studies were missing.
Question and Inclusion/ Excluded Studies Justification	Did not mention inclusion/exclusion criteria	Three of eight included studies were not about coronavirus but different types of viruses: influenza [5] and RSV [6,7]
Study design	Selection bias was developed because there was no study design in the manuscript<br>There was no description about populations, interventions, comparators	Three of eight included studies were only investigated among steroid used patients because the primary outcome were complications of steroids: psychosis [8], steroid induced diabetes mellitus [9] and osteonecrosis [10].
Statistical Methods	There was no statistical process or summary about the data from all included studies	Weak evidence of the conclusion was developed. The results of all eight included studies deviated to negative effects of steroids but there was no explanation about heterogeneity and publication bias.
Our study		
Comprehensive Search	Comprehensive literature search strategy was performed.	Most of coronavirus studies were reviewed during search process and included all studies about efficiency of steroids related to mortality.
Question and Inclusion/ Excluded Studies Justification	Precise Inclusion/exclusion criteria and selection of the study were described in methods.	Restricted all Included studies only about coronaviruses excluding other types of viruses.
Study design	Study design was explained in the manuscript.	All included studies had steroid group and non-steroid group (control) with the number of deaths as the primary outcome.
Statistical Methods	There was a statistical process combining raw data from all included studies.	Comprehensive meta-analyses were performed to combine study results to explain based on the statistical evidence. Heterogeneity and publication bias were also described.

RSV: respiratory syncytial viruses.

5. Quality Assessment of the included Studies (Table S6–7)

We performed quality assessment of each included study based on an adapted version of Newcastle-Ottawa scale [11]. In each study, we divided the selection, the comparability, and outcome part to give scores for a total of 8 points. We ranked the studies according to the score (7 or more at high quality, moderate at 4 or more and less than 6, and Low quality 3 points or less. As a result, 2/9 (22.2%) of studies were high quality, 0/9 (0.0%) were moderate, and 7/9 (77.8%) were low quality. Bias was also assessed and higher scores indicate both higher study quality and lower risk of bias.

Table S6. Quality assessment * of the cohort studies included in the meta-analysis (selection part).

Authors	Type of Study	Selection									
		Representativeness of the Exposed Cohort/Sample [a] (1)				Selection of the Non-Exposed Cohort [b] (1)			Ascertainment of Exposure [c] (1)		
		Truly representative (A, one star)	Somewhat Representative (B, one star)	Selected group (C, no star)	No description of the derivation of the cohort/sampling strategy (D, no star)	Drawn from the same community as the exposed cohort (A, one star)	Drawn from a different source (B, no star)	No description of the derivation of the non-exposed cohort (C, no star)	Description of kind and dose of steroids (A, one star)	Only described as simple steroids (B, one star)	No description (C, no star)
Li et al. (2003) [12]	Retrospective Cohort	1				1			1		
Yam et al. (2007) [13]	Retrospective Cohort		1				0		1		
Lau et al. (2009)_H † [14]	Retrospective Cohort		1				0			1	
Lau et al. (2009)_T † [14]	Retrospective Cohort		1				0			1	
Arabi et al. (2018) [15]	Retrospective Cohort	1				1			1		
Chen et al. (2006) [16]	Retrospective Cohort	1				1			1		
Wu et al. (2020) [17]	Retrospective Cohort	1				1			1		
Al Ghamdi et al. (2016) [18]	Retrospective Cohort		1				0		1		

Zhou et al. (2020) [19]	Retrospective Cohort	1	0	1

* Quality assessment scoring: 1=study met the criteria (one star); 0 = study did not meet the criteria, or not reported (no star). a A*-Representative of the steroid group (Intervention about only steroid-used group); B*-Somewhat representative of the steroid group (Intervention about not-only about steroids, Risk factor); C -Selected group, chance of bias; D-No description of the derivation of the steroid group. b A*-Reported numbers of control at baseline; B- Reported control as an exclusion criteria for steroid therapy; C-No demonstration of control at the baseline. c A*-Description about both the kind and dose of steroids was done for all steroid-used patients; B*–Description about one of the kind and dose of steroids (must 1 of 2); C-Only described as 'corticosteroids' or 'steroids' without explaining about the kind and dose. † These are the same paper (Lau, 2009) [14] which has the two subgroups: one study in Hong-Kong(H) and the other study in Toronto (T).

Table S7. Quality assessment * of the cohort studies included in the meta-analysis (Comparability/Outcome part).

Authors	Comparability		Outcome (One Star Per Item)											Total Score (All Studies = 8)	Quality
	**Comparability on the basis of the design or analysis controlled for confounders [d] (1)**		**Assessment of outcome [e] (1)**			**Was duration of follow up explicitly indicated? [f] (1)**		**Adequacy of follow-up cohorts [g] (1)**				**Statistical Test [h] (1)**			
	Study controls for relevant factors (e.g. age, sex) (A/B, one star)	Not comparable on the basis of study design or analysis (C/D/E, no star)	High assessment of outcome (A, one star)	Small asscessment of outcome (B, no star)	No description (C, no star)	Yes (A, one star)	No (B, no star)	Complete follow up reported. All subjects accounted for (A/B, one star)	Subjects lost to follow-up are discussed or are unlikely to introduce bias (C, one star)	Subjects lost to follow-up are not discussed or may introduce bias (D, no star)	No reporting of subjects lost to follow-up (E, no star)	Sufficient data (A, one star)	Inappropriate statistical test (B, no star)	Rating	
Li et al. (2003) [12]		0		0			0				0		0	3	Low
Yam et al. (2007) [13]		0		0		1					0		0	3	Low
Lau et al. (2009)_H † [14]		0		0			0				0		0	2	Low
Lau et al. (2009)_T † [14]		0		0			0				0		0	2	Low
Arabi et al. (2018) [15]		0		0			0				0		0	3	Low
Chen et al. (2006) [16]		0	1			1			1			1		7	High
Wu et al. (2020) [17]		0	1			1			1			1		7	High
Al Ghamdi et al. (2016) [18]		0		0			0				0		0	2	Low
Zhou et al. (2020) [19]		0		0			0				0		0	2	Low

* Quality assessment scoring: 1=study met the criteria (one star); 0=study did not meet the criteria, or not reported (no star). d A*-Prospective Cohort; B*-Adjusted odds ratio; C-Retrospective Cohort; D-Adjusted Odd Ratio not specified; E-nothing specified. e A*-Mortality variables were adjusted in steroid and control groups; B*-Mortality variables were not adjusted and only description about mortality with numbers; C-No description. f A*-Yes (time related steroids is described) after

exposure to patients; B–No (Information not provided). g A*-If prospective, all patients were evaluated for use of steroids during follow-up; B*-If prospective, <=10% of patients lost to follow up; C*-If retrospective, number of patients lost to follow-up or excluded is reported and <=10%; D-If retrospective or prospective, greater than 10% lost to follow up; E-If prospective or retrospective, number of patients lost to follow up not reported. h A*-Sufficient data and statistical test about steroids presented to support the primary outcome (mortality); B-The statistical test is not appropriate, not described or incomplete. † These are the same paper (Lau, 2009) [14] which has the two subgroups: one study in Hong-Kong (H) and the other study in Toronto (T).

6. Detailed description of included studies (Table S8–9)

Table S8. Detailed description about basal characteristics of included studies.

Authors	Type of Disease	Number of Hospital	Location/Nationality	Patient Group	Study Duration	Mean Age (Years)	Male	Female	Steroid	Non-Steroid	Type of Case	Subgroup Case	Subgroup Control
**Li et al. (2003) [12]**	SARS	1	Beijing/China	Non-ICU	-	-	-	-	39	4	Use of methylprednisolone	-	-
**Yam et al. (2006) [13]**	SARS	14	Hong Kong/China	ICU/Non-ICU	Mar - Oct 2003	-	553	734	1188	99	IV hydrocortisone	621	99
											IV methylprednisolone	177	
											Oral prednisolone	170	
											IV pulsed methylprednisolone	220	
**Lau et al. (2009) [14]**	SARS	-	Hong Kong/China	ICU/Non-ICU	2002-2003	-	773	970	790	953	Use of corticosteroid	-	-
			Toronto/Canada		Onset before April 22, 2003)		74	117	42	149	Use of corticosteroid	-	-
**Arabi et al. (2018)* [15]**	MERS	14	All/Saudi Arabia	ICU	Sep 2012- Oct 2015	-	213	96	151	158	Use of hydrocortisone, dexamethasone, methylprednisolone, IV pulsed methylprednisolone	-	-
**Chen et al. (2006) [16]**	SARS	-	Guangzhou/China	ICU/Non-ICU	Dec 2002- Jun 2003	34.74 ± 13.31	129	272	268	133	Use of hydrocortisone, prednisolone, methylprednisolone	-	-
**Wu et al. (2020) [17]**	COVID-19	1	Wuhan/China	ICU/Non-ICU	Dec 25, 2019 – Jan 26, 2020	51	128	73	151	158	Use of methylprednisolone	-	-
**Al Ghamdi**	MERS	1	Jeddah/Saudi Arabia	ICU/Non-ICU	Jan-Dec 2014	54	40	11	5	46	Use of corticosteroid among survivors	2	3

**et al. (2016) [18]**													
**Zhou et al. (2020) [19]**	COVID-19	2	Wuhan/China	ICU/Non-ICU	By Jan 31, 2020	56	119	72	57	134	Use of corticosteroid	-	-

SARS: Severe acute respiratory syndrome, MERS: Middle East respiratory syndrome, COVID-19: Coronavirus disease 19, ICU: intensive care unit, ARDS: acute respiratory distress syndrome, (-): no information. * This paper is also described in Russell (2020) [4] as references.

Table S9. Detailed description about steroids of included studies.

Authors	Steroid Type	Steroid Dose in Article	Mean Duration of Steroids, Day	Mean Duration between Onset of Illness and Steroid Initiation, d	Primary Endpoint of the Study	Mortality in Steroids	Mortality in Non-Steroids	Description about the Steroids in the Study	Type of Case	Conclusion
**Li et al. (2003) [12]**	Methylprednisolone	170.82 +/- 15.89 (Day 1) max dose 291.44 +/- 37.63 (Day1)	-	-	Treated SARS	1	0	Sub-pulse dosage of MP was effective for most SARS patients. Those who were less responsiveness might due to their poor sensitiveness to corticosteroids instead of SARS severity	**Use of steroid**	**Helpful**
**Yam et al. (2006) [13]**	IV hydrocortisone	13200mg/total	19	4	Treated SARS	202	28	Among four corticosteroid groups studied, mortality was lowest in the low-dose oral prednisolone (Group P) and high-dose methylprednisolone (Group MP) groups.	**Combination therapy - corticosteroid and ribavirin**	**Helpful**
	IV methylprednisolone	11350mg/total	21	5						
	Oral prednisolone	7020mg/total	15	5						
	IV pulsed methylprednisolone	17560mg/total	19	6						
**Lau et al. (2009) [14]**	-	-	-	-	Treated SARS	108	193	The combination of ribavirin and corticosteroids has no significant beneficial effect in the treatment of SARS.	**Combination therapy - corticosteroid and ribavirin**	**Not-Helpful**
						6	19			
**Arabi et al. (2018)* [15]**	Hydrocortisone Dexamethasone Methylprednisolone Prednisolone	-	7.0 (4.0–14.0) (case 10.0 (4.0-19.0) control 7.0(4.0-12.0))	10.0 (7.0–17.0) Illness to steroid	Treated MERS	117	92	Corticosteroid therapy in patients with MERS was not associated with a difference in mortality after adjustment for time varying confounders but was associated with delayed MERS coronavirus RNA clearance.	**Use of steroid**	**Inconclusive**
**Chen et al. (2006) [16]**	Hydrocortisone, Methylprednisolone, prednisolone,	Total Median MP 1868.06mg (1723.6 mg vs	-	5.01+/-3.48 (5.00 +/- 3.52 vs 5.04 +/- 3.12, p=0.961)	Treated SARS	18	7	Proper use of corticosteroid in confirmed critical SARS resulted in lowered mortality and shorter hospitalization stay, and was not associated with significant	**Use of steroid**	**Helpful**

		3874.42mg, p=0.011)						secondary lower respiratory infection and other complications.		
**Wu et al. (2020) [17]**	-	-	-	-	Treated COVID-19	23	39	Among patients with ARDS, treatment with methylprednisolone decreased the risk of death (HR, 0.38; 95%CI, 0.20-0.72).	**Use of steroid**	**Helpful**
**Al Ghamdi et al. (2016) [18]**	Hydrocortisone	-	-	-	Treated MERS	3	16	In this retrospective cohort, interferon beta and mycophenolate mofetil treatment were predictors of increased survival in the univariate analysis. (Steroid is not a predictive factor of survival)	**Use of steroid**	**Inconclusive**
**Zhou et al. (2020) [19]**	-	-	12	-	Treated COVID-19	26	28	High-dose corticosteroid use might have also contributed to the poor clinical outcomes in some patients.	**Use of steroid**	**Inconclusive**

SARS: Severe acute respiratory syndrome, MERS: Middle East respiratory syndrome, COVID-19: Coronavirus disease 19, ICU: intensive care unit, ARDS: acute respiratory distress syndrome, MP: methylprednisolone, HR: hazard ratio, CI: confidence interval, (-): no information. * This paper is also described in Russell (2020) [4] as references.

7. Funnel plots (Figure S1–3)

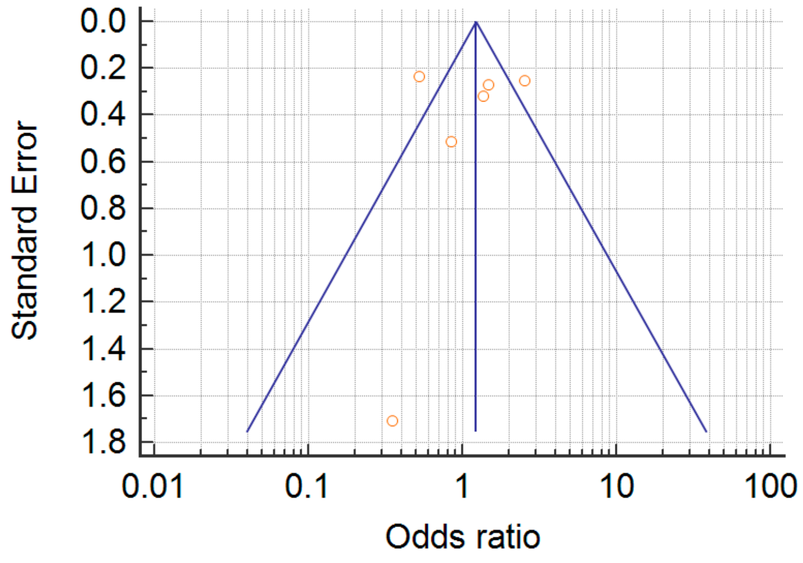

Figure S1. Funnel plot for meta-analysis of association between steroids and mortality of studies about intervention (in total).

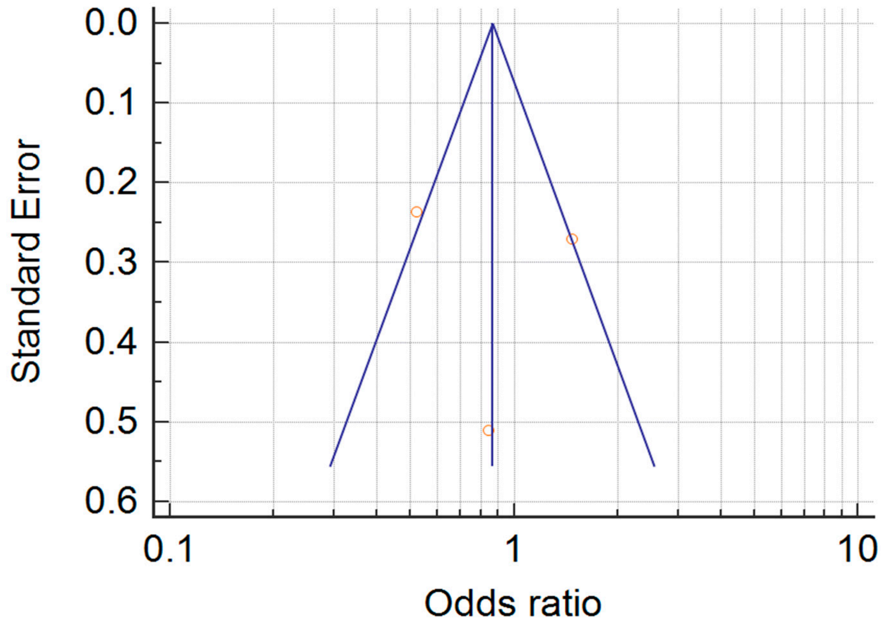

Figure S2. Funnel plot for meta-analysis of association and mortality of studies about steroids as an add-on therapy for ribavirin.

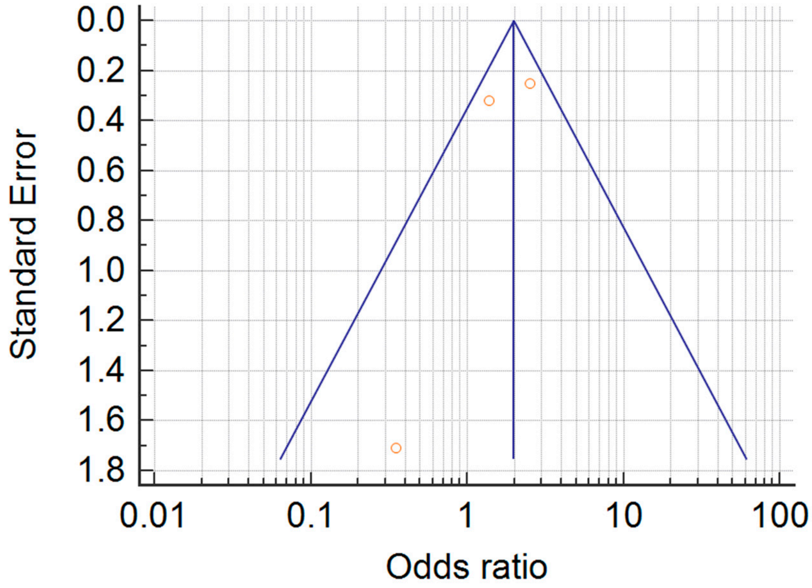

Figure S3. Funnel plot for meta-analysis of association between steroids and mortality of studies about steroids itself comparing non-steroid group.

8. Subset Analyses (Figure S4)

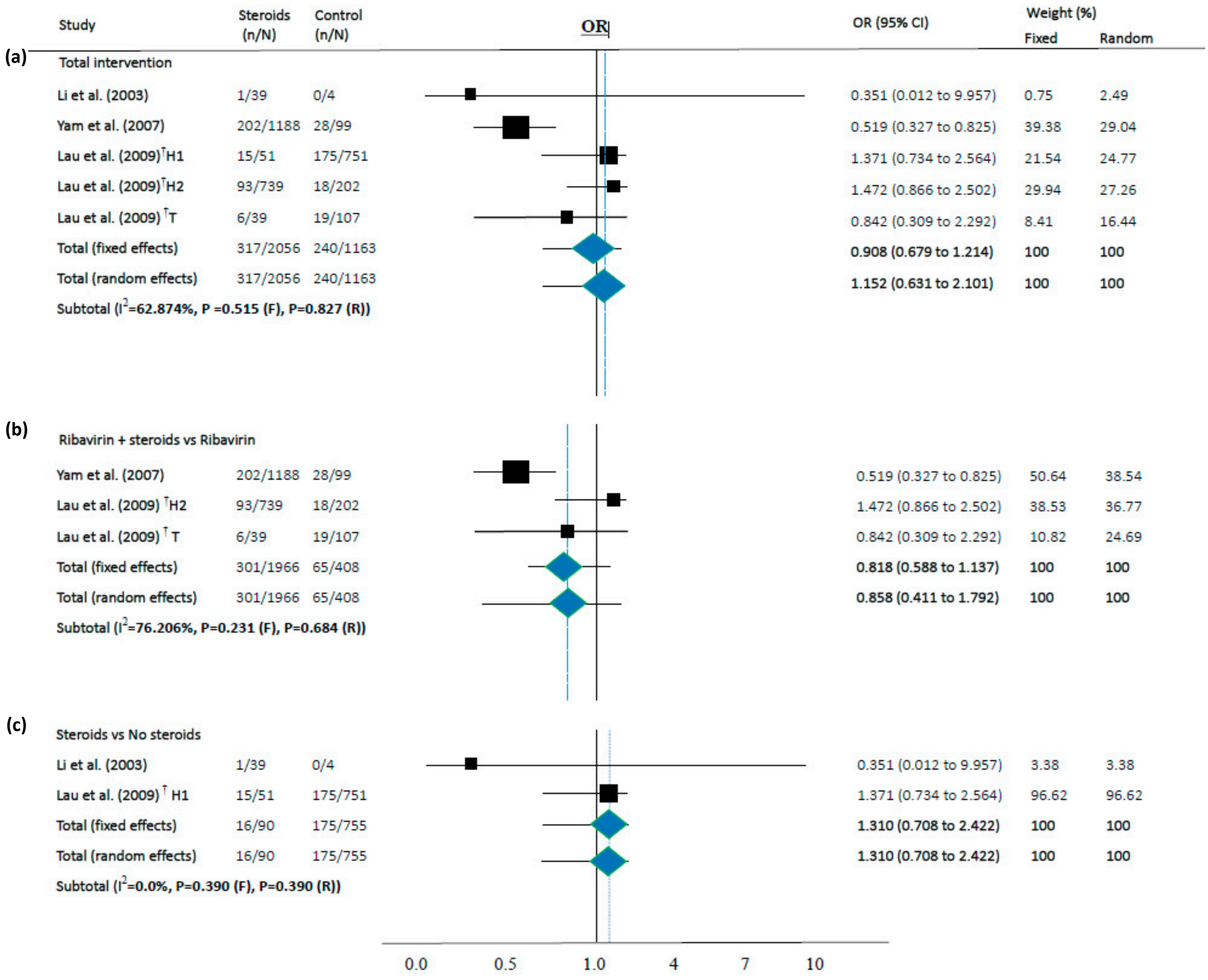

Figure S4. Association between steroids and mortality of SARS studies about "intervention". Studies are presented as country study (study [year]). The data are presented for total SARS studies about intervention (**a**), steroids as an add-on therapy for ribavirin (**b**), and steroids itself comparing non-steroid group (**c**). † These are the same paper (Lau (2009) [14]) which has the two subgroups: one study conducted in Hong-Kong (H1 and H2) and the other study in Toronto (T).

9. References.

1. Moher, D.; Liberati, A.; Tetzlaff, J.; Altman, D.G. PRISMA Group. Preferred reporting items for systematic reviews and meta-analyses: The PRISMA statement. *J. Clin. Epidemiol.* **2009**, *62*, 1006–1012.
2. Stockman, L.J.; Bellamy, R.; Garner, P. SARS: Systematic review of treatment effects. *PLoS Med.* **2006**, *3*, e343.
3. Zhao, R.; Wang, H.; Wang, X.; Feng, F. Steroid therapy and the risk of osteonecrosis in SARS patients: A dose-response meta-analysis. *Osteoporos. Int.* **2017**, *28*, 1027–1034.
4. Russell, C.D.; Millar, J.E.; Baillie, J.K. Clinical evidence does not support corticosteroid treatment for 2019-nCoV lung injury. *Lancet* **2020**, *395*, 473–475.
5. Ni, Y.N.; Chen. G.; Sun, J.; Liang, B.M.; Liang, Z.A. The effect of corticosteroids on mortality of patients with influenza pneumonia: a systematic review and meta-analysis. *Crit. Care* **2019**, *23*, 99.
6. McGee, S.; Hirschmann. J. Use of corticosteroids in treating infectious diseases. *Arch Intern Med* **2008**, *168*, 1034–1046.
7. Corneli, H.M.; Zorc, J.J.; Mahajan, P.; Shaw, K.N.; Holubkov, R.; Reeves, S.D.; Richard M.; Ruddy, B.M.; Nelson, K.A.; Bregstein, J.S.; et al. A multicenter, randomized, controlled trial of dexamethasone for bronchiolitis. *N. Engl. J. Med.* **2007**, *357*, 331–339.
8. Lee, D.T.; Wing, Y.K.; Leung, H.C.; Sung, J.J.; Ng, Y.K.; Yiu, G.C.; Chen, R.Y.; Chiu, H.F. Factors associated with psychosis among patients with severe acute respiratory syndrome: a case-control study. *Clin. Infect. Dis.* **2004**, *39*, 1247–1249 (in Chinese).
9. Xiao, J.Z., Ma, L.; Gao, J.; Yang, Z.J.; Xing, X.Y.; Zhao, H.C.; Jiao, J.S.; Li, G.W. Glucocorticoid-induced diabetes in severe acute respiratory syndrome: the impact of high dosage and duration of methylprednisolone therapy. *Zhonghua Nei Ke Za Zhi.* **2004**, *43*, 179–182 (in Chinese).
10. Li, Y.M.; Wang, S.X.; Gao, H.S.; Wang, J.G.; Wei, C.S.; Chen, L.M.; Hui, W.L.; Yuan, S.L.; Jiao, Z.S.; Yang, Z., et al. Factors of avascular necrosis of femoral head and osteoporosis in SARS patients' convalescence. *Zhonghua Yi Xue Za Zhi* **2004**, *84*, 1348–1353 (in Chinese).
11. Wells, G.A.; Shea, B.; O'Connell, D.; Peterson, J.; Welch, V.; Losos, M.; Tugwell, P.; Wells, G.A.; O'Connell, D.; Shea, B. The Newcastle-Ottawa Scale (NOS) for assessing the quality of nonrandomised studies in meta-analyses. http://www.ohri.ca/programs/clinical_epidemiology/oxford.asp (accessed on 28 March 2020).
12. Li, N.; Ma, J.; Nie, L.; Li, H.; Que, C.; Gao, Z.; Wang, G.; Xu, X.; Lu, H.; Wang, G. Retrospective analysis of the corticosteroids treatment on severe acute respiratory syndrome (SARS). *Beijing Da Xue Xue Bao Yi Xue Ban* **2003**, *35* (Suppl:16–8) (in Chinese).
13. Yam, L.Y.; Lau, A.C.; Lai, F.Y.; Shung, E.; Chan, J.; Wong, V.; Hong Kong Hospital Authority SARS Collaborative Group (HASCOG). Corticosteroid treatment of severe acute respiratory syndrome in Hong Kong. *J. Infect.* **2007**, *54*, 28–39.
14. Lau, E.H.; Cowling, B.J.; Muller, M.P.; Ho, L.M.; Tsang, T.; Lo, S.V.; Louie, M.; Leung, G.M. Effectiveness of Ribavirin and Corticosteroids for Severe Acute Respiratory Syndrome. *Am J Med* **2009**, *122*, 1150.e11–1150.e21.
15. Arabi, Y.M.; Mandourah, Y.; Al-Hameed, F.; Sindi, A.A.; Almekhlafi, G.A.; Hussein, M.A.; Jose, J.; Pinto, R.; Al-Omari, A.; Kharaba, A.; et al. Corticosteroid Therapy for Critically Ill Patients with Middle East Respiratory Syndrome. *Am. J. Respir. Crit. Care Med.* **2018**, *197*, 757–767.
16. Chen, R.C.; Tang, X.P.; Tan, S.Y.; Liang, B.L.; Wan, Z.Y.; Fang, J.Q.; Zhong, N. Treatment of Severe Acute Respiratory Syndrome with glucosteroids: The Guangzhou experience. *Chest.* **2006**, *129*, 1441–1452.
17. Wu, C.; Chen, X.; Cai, Y.; Xia, J.; Zhou, X.; Xu, S.; Huang, H.; Zhang, L.; Zhou, X.; Du, C.; et al. Risk Factors Associated With Acute Respiratory Distress Syndrome and Death in Patients With Coronavirus Disease 2019 Pneumonia in Wuhan, China. *JAMA Intern. Med.* **2020**, *180*, 934–943
18. Al, Ghamdi.M.; Alghamdi, K.M.; Ghandoora, Y.; Alzahrani, A.; Salah, F.; Alsulami, A.; Bawayan, M.F.; Vaidya, D.; Perl, T.M.; Sood, G. Treatment outcomes for patients with Middle Eastern Respiratory Syndrome Coronavirus (MERS CoV) infection at a coronavirus referral center in the Kingdom of Saudi Arabia. *BMC Infect. Dis.* **2016**, *16*, 174.
19. Zhou, F.; Yu, T.; Du, R.; Fan, G.; Liu, Y.; Liu, Z.; Xiang, J.; Wang, Y.; Song, B.; Gu, X.; et al. Clinical course and risk factors for mortality of adult inpatients with COVID-19 in Wuhan, China: A retrospective cohort study. *Lancet* **2020**, *395*, 1054–1062.
